# Role of the chaperonin TCP-1 ring complex in protein aggregation and neurodegeneration

**DOI:** 10.3389/fnmol.2025.1617771

**Published:** 2025-07-07

**Authors:** Vanlalrinchhani Varte, Diego E. Rincon-Limas

**Affiliations:** ^1^Department of Neurology, McKnight Brain Institute, Norman Fixel Institute for Neurological Diseases, University of Florida, Gainesville, FL, United States; ^2^Department of Neuroscience, Center for Translational Research in Neurodegenerative Disease, University of Florida, Gainesville, FL, United States; ^3^Genetics Institute, University of Florida, Gainesville, FL, United States

**Keywords:** aggrephagy, Alzheimer, amyloid beta, CCT complex, chaperonin, polyQ, tau, TRiC

## Abstract

The chaperonin TCP-1 ring complex (TRiC), also known as chaperonin-containing TCP-1 (CCT) complex, plays a crucial role in protein folding and quality control within the cell. Comprising eight distinct subunits (CCT1 - CCT8), TRiC assists in the folding of a wide range of client proteins, ensuring their proper conformation and functionality. This mini review explores the assembly, structure, and cellular functions of TRiC and discusses its involvement in protein aggregation and neurodegenerative diseases. We emphasize the emerging role of CCT2 in modulating the formation of abnormal amyloid aggregates, including amyloid beta, tau, and polyglutamine (polyQ) deposits, which are central to the pathogenesis of various neurological conditions. Lastly, we provide evidence supporting the neuroprotective role of CCT2 *in vivo* and also highlight therapeutic implications and key unresolved questions in the field, offering a foundation for new research opportunities.

## Introduction

Proteins must fold correctly to function and maintain cellular homeostasis. To achieve correct folding, cells rely on a class of proteins known as molecular chaperones, which assist in the folding, assembly, and transport of proteins. One of the most important chaperone machineries mediating proteostasis is the chaperonin TCP-1 ring complex (TRiC), also known as Chaperonin-containing TCP-1 (CCT) complex (Grantham, 2020). TRiC is a large (~1 MD), multi-subunit complex found in the cytosol and is highly conserved among eukaryotes (Kim et al., 2024). Unlike other chaperones, TRiC specializes in the folding of large, complex cytosolic proteins, including actin and tubulin, which are fundamental components of the cytoskeleton (Grantham et al., 2006; Vallin and Grantham, 2019; Willison, 2018). Interestingly, 10 % of the cytosolic protein substrates, such as cytoskeletal proteins, cell cycle regulators, and aggregation-prone proteins, are folded by TRiC (Gestaut et al., 2019; Jin et al., 2019b).

TRiC is comprised of two octameric rings stacked back-to-back to form a barrel-like structure and each ring contains eight subunits (CCT1–8). Each ring retains the same component order (CCT 2–4–1-3-6-8-7-5) with a two-fold symmetry axis, and the structure exhibits asymmetry in the chaperone folding chamber (Leitner et al., 2012). Each subunit has apical and equatorial domains, which are in charge of substrate recognition/binding and ATP binding, respectively. An intermediate domain functions as hinge between these two domains and facilitates movements associated with conformational changes caused by ATP cycling (Kim et al., 2024). Thus, coordinated ATP-driven conformational cycles and proper communication between the apical and equatorial domains are critical for protein folding (Gestaut et al., 2019; Jin et al., 2019b). Despite having almost identical ATPase domains, the polypeptide-binding portions of each TRiC subunit have undergone significant divergence over evolution to produce substrate-binding specificity (Kim et al., 1994). Indeed, TRiC exhibits subunit selectivity regarding ring closure, ATP consumption, and complex assembly (Jin et al., 2019a; Reissmann et al., 2012; Zang et al., 2016).

### Role of TRiC in protein folding and quality control

As soon as polypeptides leave the ribosomal exit tunnel, a complex network of molecular chaperone proteins assist in their folding. These molecular chaperones attach to their folding intermediates to stop polypeptides from aggregating and, in certain situations, actively assist them in folding through cycles of binding and release (Young et al., 2004).

**Figure 1:**
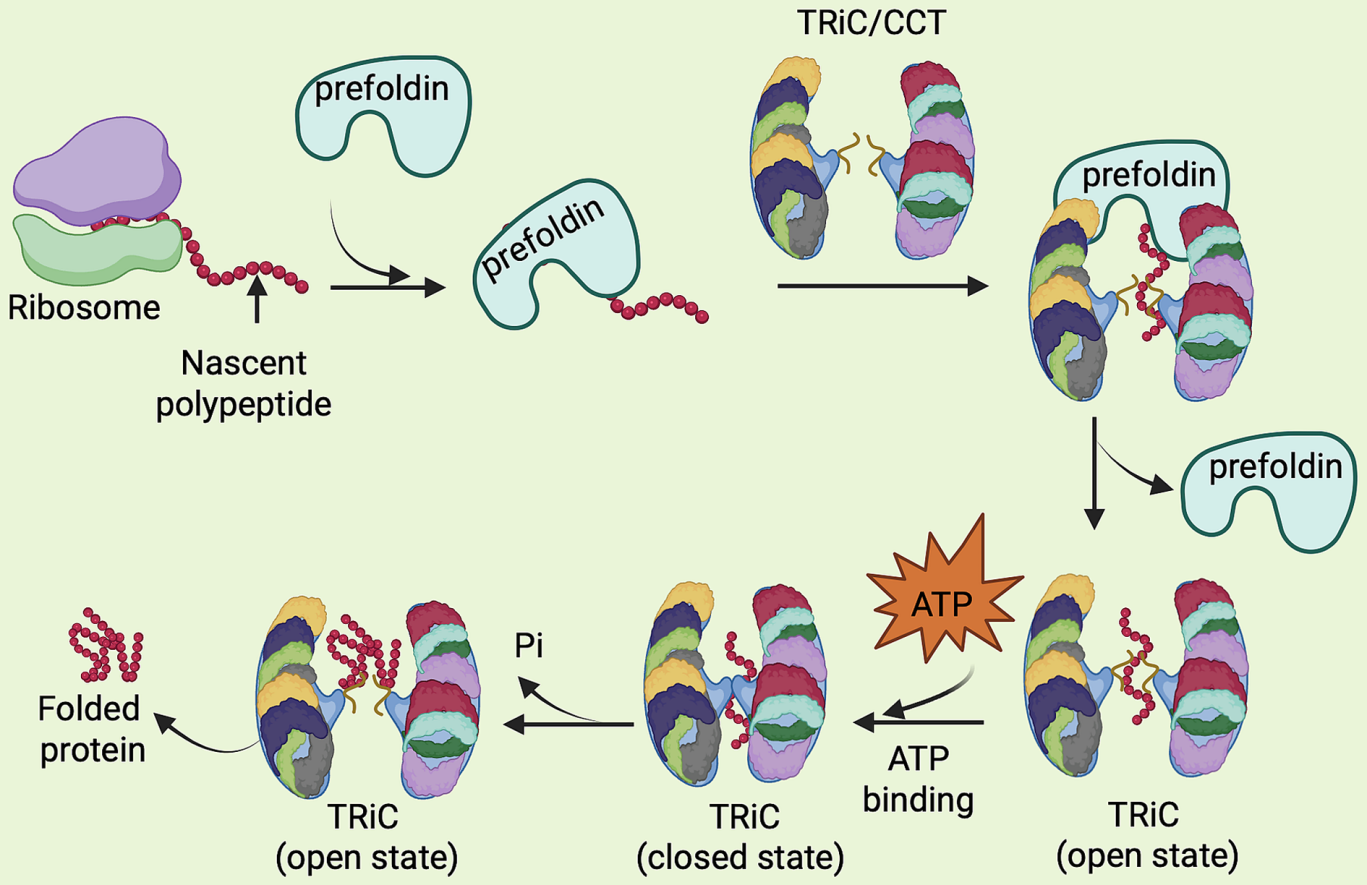
Functional representation of the TRiC/CCT complex in protein folding. Newly synthesized proteins are initially captured by the chaperone prefoldin, which delivers their folding intermediates to the TriC/CCT complex to complete proper folding. ATP hydrolysis triggers a conformational change in TRiC from an open to closed state, forming a protective chamber that facilitates protein folding. Once folding is complete, the release of inorganic phosphate (Pi) reopens the complex, allowing the folded protein to exit. The illustration shows a side view of TRiC, with each CCT subunit represented in a distinct color.

The role of TRiC in this process involves a series of intricate steps. Upon binding to unfolded polypeptides delivered by the prefoldin/Gim1-6 complex (GimC), TRiC forms a double-ring structure that provides an enclosed, ATP-driven environment where proteins can fold without interference from the surrounding cellular environment. This process is highly dynamic, with ATP hydrolysis driving conformational changes in the TRiC complex, including specific interactions between apical domains that stabilize a stiffer and more compact open conformation (Meyer et al., 2003). Subsequent ATP hydrolysis causes the apical domains to undergo conformational rotation. This rotation completes two essential functions: the lid is closed, and the bound substrate is released into the central chamber, which now takes on a highly polar and charged environment that facilitate the proper folding of the substrate (Kim et al., 2024). When TRiC substrates reach their native and intended conformations, the chaperonin must coordinate their release so that they can carry out their biological roles. Additional changes in conformation brought on by ATP hydrolysis cause TRiC to release its cargo, preventing the formation of misfolded proteins (Gestaut et al., 2019; Gestaut et al., 2022; Wang et al., 2023) (Figure 1). Thus, TriC is essential not only to ensure proper protein folding, but also for maintaining cellular integrity and preventing proteotoxic stress (Gestaut et al., 2019; Young et al., 2004).

### TRiC and protein aggregation

TRiC substrates are often big as the size of the folding chamber is limited to about 70 kDa. These substrates are generally hydrophobic polypeptides with complex structures, slow folding kinetics, and prone to aggregation, such as *β*-strand-containing proteins (Camasses et al., 2003; Kubota et al., 2006; Rommelaere et al., 1999; Yam et al., 2008). The hydrophobic regions of these proteins are normally hidden, but their abnormal exposure can trigger protein aggregation. This is because the exposed areas may interact with other misfolded proteins, leading to the formation of insoluble aggregates.

One of the primary mechanisms behind TRiC/CCT dysfunction and protein aggregation is the failure of the ATPase cycle within the complex. As described above, ATP hydrolysis drives conformational changes in the TRiC/CCT rings, which are necessary for its function in protein folding. If the ATPase activity is impaired due to mutations in the subunits of the TRiC/CCT complex or disturbances in its assembly, the chamber of the complex may fail to open and close properly, preventing the efficient folding of substrates (Reissmann et al., 2012; Roy et al., 2023). This can lead to the accumulation of unfolded or partially folded proteins that are prone to aggregation, particularly under conditions of cellular stress that require increased protein folding such as in neurodegenerative diseases. In this context, a malfunction of TRiC/CCT induces the formation of toxic aggregates, contributing to the progressive loss of neuronal integrity and function.

### Polyglutamine (polyQ) proteins and TRiC

Polyglutamine (polyQ) disorders, such as Huntington’s disease (HD), are characterized by the aggregation of proteins containing an expanded polyQ tract. As with all other amyloidogenic proteins, polyQ-expanded proteins consistently form fibrillar aggregates with a “cross-*β*” core and a broad β-sheet structure (Chiti and Dobson, 2006). CCT/TRiC was initially identified as a possible regulator of protein aggregation through a *C. elegans* RNAi screen for genes that cause aggregation of polyglutamine tracts upon silencing. Interestingly, among the hits within the “protein folding” functional category, six CCT subunits, along with Hsp70 and DnaJ, were found in this screen (Nollen et al., 2004). Subsequent studies also identified PolyQ-expanded huntingtin as a TRiC substrate (Behrends et al., 2006; Kitamura et al., 2006; Tam et al., 2006) and two groups demonstrated that TRiC regulates cell death, partially colocalizes with huntingtin aggregates and alters their shape (Behrends et al., 2006; Kitamura et al., 2006). Consistently, when CCT subunits were knocked-down in polyQ35-expressing worms, there was a significant decrease in the mobility of these animals, but not in the wild-type worms (Brehme et al., 2014). Moreover, reduction of all subunits except CCT5 in HeLa cells expressing Htt-exon1 (Q78)-GFP increased the formation of aggregates (Brehme et al., 2014), suggesting that enhancing TRiC activity may have potential therapeutic effects.

**Figure 2:**
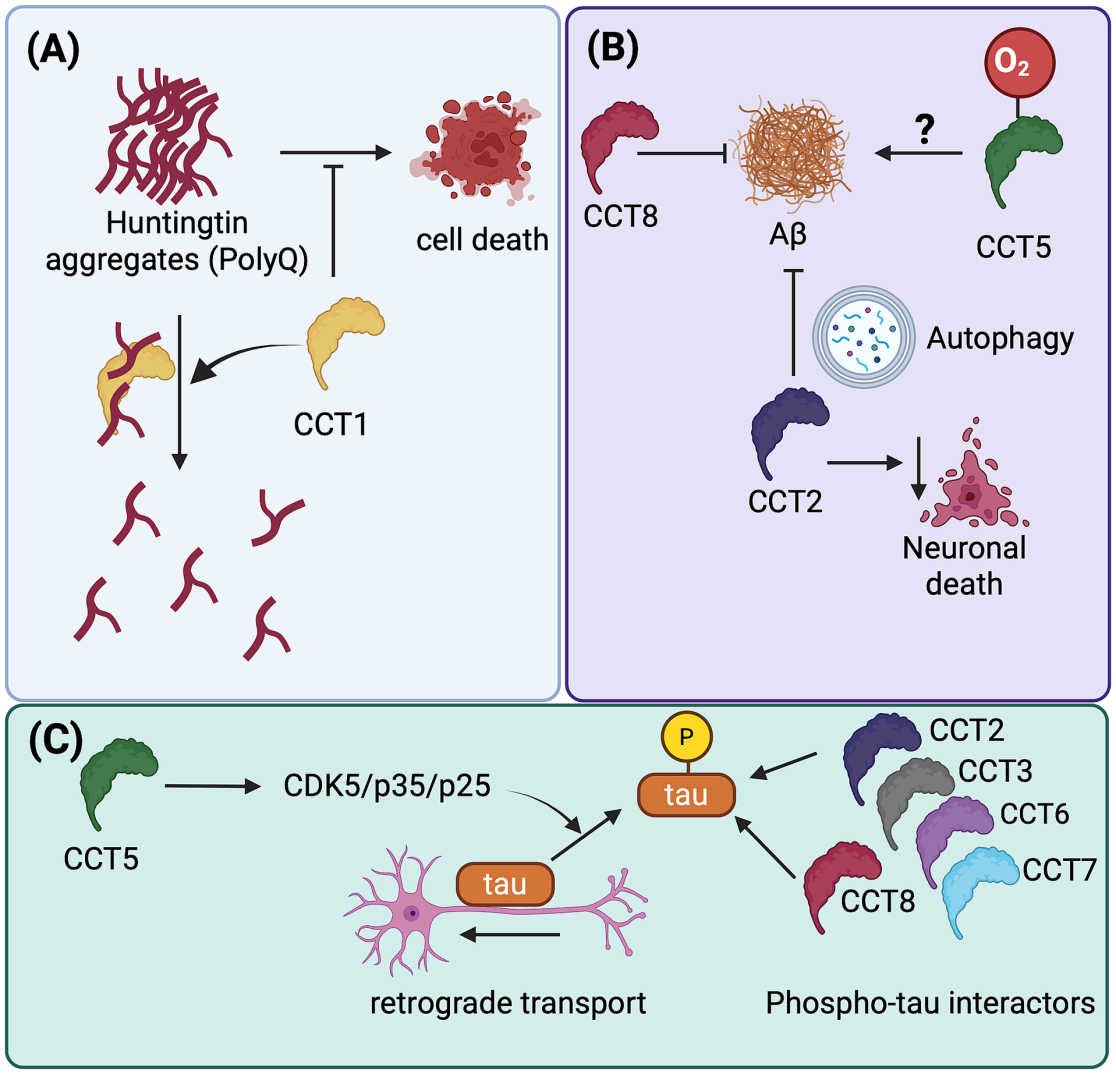
Functional interactions of selected TriC subunits with expanded huntingtin, amyloid beta and tau. **(A)** While partially colocalizing with huntingtin aggregates, CCT1 decreases polyglutamine aggregation and also alleviates cell death. **(B)** CCT8 suppresses amyloid beta aggregates, and oxidation of CCT5 might cause excessive protein folding and subsequent protein aggregation by impairing the overall TriC activity. In addition, CCT2 clears amyloid by autophagy and reduces neuronal death. **(C)** Over-expression of CCT5 increases tau phosphorylation via CDK5/p35/p25 and separates tau from the microtubule, which enhances axonal transport. The indicated CCT subunits were found to be associated with phosphorylated tau through proximity proteomics, but the relevance of these interactions is unknown and license from BioRender.com.

The role of individual CCT subunits was evidenced when CCT1 overexpression was found to decrease expanded Htt aggregation and extend viability in neuronal cells (Sontag et al., 2013; Tam et al., 2006). Additional subunit-specific studies found that the purified apical domain of CCT1, but not the apical domains from CCT3 and CCT7, interacted physically with the Htt Exon 1 and was sufficient to inhibit polyQ aggregation in a dose-dependent manner (Sontag et al., 2013, Tam et al., 2006). Recent evidence also found that CCT2 interacts with Htt fragments and with mutant Ataxin 3, another protein linked to polyglutamine disorders (Pavel et al., 2016). Regardless of these interactions, other subunits are also involved in folding and aggregation of polyglutamine tracts. Reduction of CCT6 function, for instance, promotes Htt aggregation and toxicity, while CCT8 overexpression extends *C. elegans* lifespan and restores homeostatic deficiencies in worms expressing mutant PolyQ tracts (Noormohammadi et al., 2016). Taken together, all these observations provide insights into how specific subunits contribute to the overall activity of the TRiC complex (Kitamura et al., 2006; Spiess et al., 2004) (Figure 2A) and its role in polyglutamine diseases.

### Amyloid beta and TRiC

Amyloid beta (Aβ) is an aggregation-prone peptide that accumulates in the Alzheimer’s disease (AD) brain and is generated through proteolytic cleavage of the Amyloid Precursor Protein (APP). Earlier genome-wide association studies uncovered the potential role of TRiC/CCT in AD (Khabirova et al., 2014). However, we were among the first to directly demonstrate that CCT2 is significantly reduced in AD brains using a bottom-up proteomics approach (Minjarez et al., 2016). In addition, an RNAi screen in Aβ-expressing *C. elegans* confirmed the functional involvement of the TRiC/CCT complex *in vivo* (Khabirova et al., 2014). In this screening, several CCT subunits emerged as suppressors of Aβ toxicity (Khabirova et al., 2014), with CCT1 and CCT8 reducing the Aβ-induced paralysis phenotype (Khabirova et al., 2014). In addition, CCT2 was identified as a potential biomarker with diagnostic value in a microarray study to find genes differentially expressed in AD patients (Liu et al., 2020). Here, CCT2 was found to be considerably downregulated in AD subjects and linked to autophagic clearance of Aβ, according to bioinformatics analysis. CCT2 was also found to have a strong negative correlation with neuronal death and a strong positive correlation with several autophagy-related pathways. Ma et al. (2022a) investigated the connection between CCT2 and autophagy-related genes, and found that CCT2 expression had a negative correlation with BAX, MAPK3, ITGB4, ATG16L2, and ERBB2, and a positive correlation with MAPK8, HSPA8, NCKAP1, RAB11A, and RAB1A (Ma et al., 2022a). This suggest that CCT2-mediated autophagy might protect against Aβ toxicity by promoting the clearance of misfolded Aβ and maintaining protein homeostasis. On the other hand, Koopman and Rüdiger examined the amounts of seven TRiC/CCT subunits in the cytoplasm of the Alzheimer brain and found no discernible changes in the levels of any TRiC/CCT subunits, with CCT2 in the entorhinal cortex showing just 10% change and CCT7 showing no change at all in the motor cortex (Koopman and Rüdiger, 2022). Regardless of this discrepancy, another study demonstrated that the protein CCT5 is selectively carbonylated in the early phases of AD. Comparing AD mice to control animals, the oxidation level of CCT5 was noticeably higher in the AD mice. The increased oxidation caused a loss of CCT5 protein folding function, resulting in incorrect protein folding and subsequent protein aggregation (Shen et al., 2015) (Figure 2B). In closing, the convergence of molecular, genetic and proteomic evidence highlights the role of the TRiC/CCT complex as a critical regulator of Aβ homeostasis.

### Tau protein and TriC

**Figure 3:**
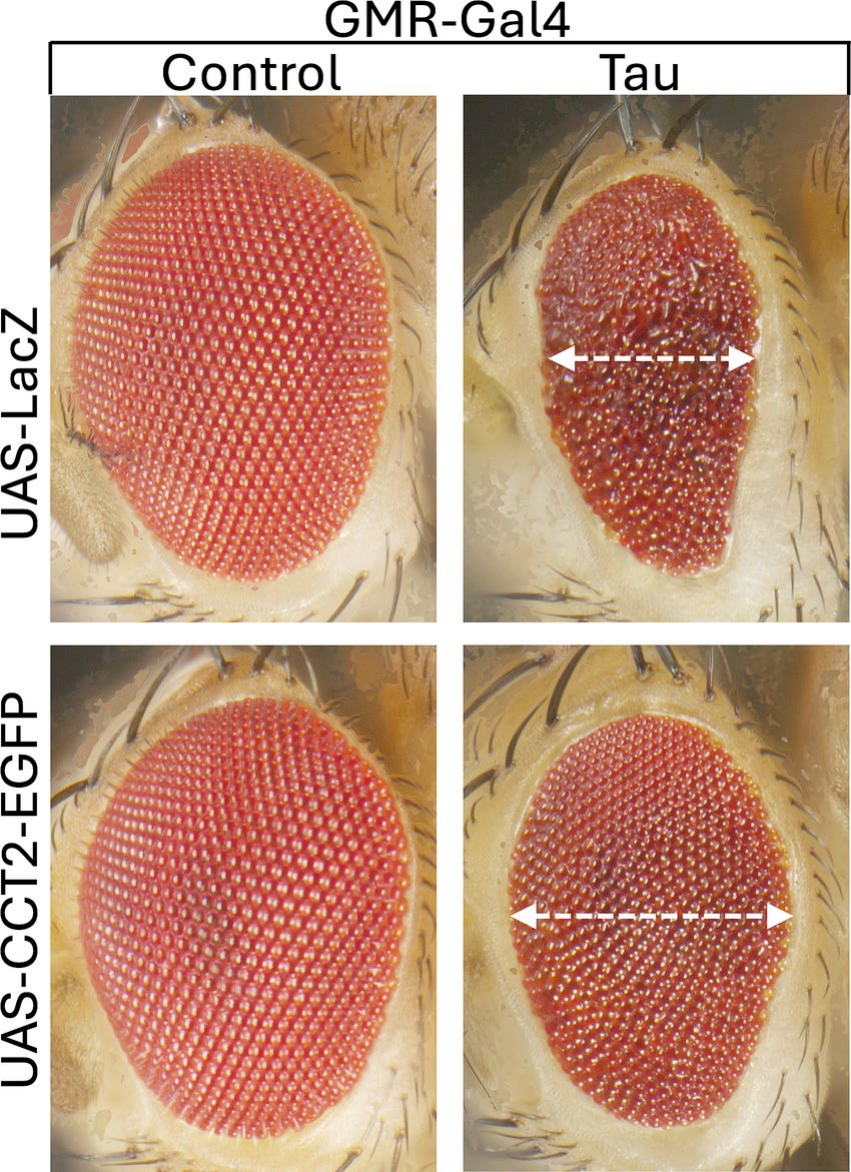
Overexpression of CCT2 alleviates tau-induced toxicity in *Drosophila*. Images show the external eye morphology of flies expressing the indicated transgenes under control of the eye-specific GMR-Gal4 driver. Control flies expressing LacZ (BDSC8529) or EGFP-tagged CCT2 (BDSC53754) alone display normal eye structure. In contrast, expression of human tau leads to a marked reduction in eye size (dotted line) and severe disruption of the retinal surface, consistent with tau-mediated toxicity (Minjarez et al., 2016). Co-expression of CCT2 with tau partially restores eye size and improves retinal organization, suggesting a protective role for CCT2 against tau toxicity and license from BioRender.com.

Tau protein aggregation is another hallmark of neurodegenerative diseases, including AD and tauopathies. Recent research indicates that CCT2 interacts with tau and triggers aggrephagy of tau aggregates (Ma et al., 2022b). This suggests that dysregulation of TRiC function may promote the formation of pathological tau aggregates, contributing to disease progression. Interestingly, aggrephagy, a selective degradation mechanism, can specifically break down hyperubiquitinated and seed-competent/aggregated tau (Tangavelou and Bhaskar, 2024). Aggrephagy receptors, such as p62 (SQSTM1/Sequestosome 1), the Next to BRCA1 gene 1 protein (NBR1), the Tax1 binding protein 1 (TAX1BP1), Optineurin (OPTN), and the Toll-interacting protein (TOLLIP), can identify protein aggregates in the aqueous phase. For the purpose of clearing protein aggregates through aggrephagy, these receptors have an LC3-interacting region motif (LIR) and a ubiquitin-binding domain (UBA) that engage with the autophagy protein LC3 and ubiquitinated protein aggregates cargo, respectively (Lamark and Johansen, 2012; Zhang and Klionsky, 2022). Interestingly, the TRiC subunit CCT2 can also identify solid protein aggregation (Ma et al., 2022b; Sun et al., 2018). Through its apical domain, CCT2 can engage with either ubiquitinated or non-ubiquitinated protein aggregation cargo to facilitate the removal of solid protein aggregates (Ma et al., 2022b). The recruitment of the autophagy receptor p62 to tau aggregates, where it binds to K63-linked polyubiquitin chains, promotes the segregation of these aggregates into larger condensates via p62-ubiquitin-mediated liquid–liquid phase separation (LLPS) (Danieli and Martens, 2018; Sun et al., 2018; Tan et al., 2008; Zaffagnini et al., 2018). Therefore, to effectively remove protein aggregates via aggrephagy, the sequential action of recruiting additional SQSTM1-like receptors (SLRs), such as NBR1 and TAX1BP1, is essential (Ferrari et al., 2024; Turco et al., 2021). Taken together, these findings support a role for CCT2 in promoting the lysosomal clearance of tau aggregates, which may underlie its protective effect against tau toxicity in the *Drosophila* eye (Figure 3). On the other side, it was recently reported that CCT5 overexpression contributes to CCT-induced tau phosphorylation by increasing CDK5/p35/p25 levels (Chen et al., 2018). CCT5 regulates retrograde axonal transport of brain-derived neurotrophic factor (BDNF) via the cyclin-dependent kinase 5 (CDK5) pathway. This effect is abolished by Roscovitine, a selective CDK5 inhibitor. However, CCT5-induced tau phosphorylation leads to the separation of tau from microtubules and prevents tau-induced microtubule bundles from forming (Chen et al., 2018) (Figure 2C). This is relevant because excessive non-phosphorylated tau has been found to impair axonal transport, probably due to stronger binding of tau to microtubules (Talmat-Amar et al., 2011). Thus, it is possible that a delicate balance between phosphorylated and non-phosphorylated tau species is critical to provide a more favorable track for motor proteins to move through the microtubules. This surprising finding highlights another mechanism by which CCT subunits regulate neuronal function. More recently, a proximity proteomics study aimed at identifying proteins associated with phospho-tau aggregates found that CCT2, CCT3, CCT6, CCT7 and CCT8 were enriched in the “protein folding” cluster (Figure 2C) (Morderer et al., 2025). However, the functional relevance of this finding remain to be elucidated.

### The role of CCT2 in modulating protein disaggregation

CCT2 is primarily known for its function as a molecular chaperone (Zhao et al., 2024). In addition to this canonical function, CCT2 plays a new role as an autophagy receptor in aggrephagy, helping to eliminate proteins that are prone to aggregation (Ma et al., 2022b). The discovery of CCT2 as an autophagy receptor responsible for clearing solid protein aggregates has transformed our understanding of protein degradation pathways (Ma et al., 2022b; Ma et al., 2022c), as discussed below.

Regardless of cargo ubiquitination, CCT2 interacts with aggregation-prone proteins and uses a non-classical V-LC3- interacting region (VLIR motif) to attach to autophagosome marker Atg8-family members (Da Silva et al., 2024). CCT2-mediated aggrephagy is a novel mechanism for the removal of aggregation-prone proteins because canonical autophagy receptors such as SQSTM1, NBR1 and TAX1BP1 are not required. Furthermore, CCT2 is independent of chaperone-mediated autophagy (CMA), the mechanism through which the lysosome degrades proteins (Bourdenx et al., 2021; Da Silva et al., 2024). When the chaperonin complex is exhausted or saturated by too many misfolded proteins, a portion of it disassembles and CCT2 turns into a monomer, exposing the VLIR motif and facilitating the binding of Atg8-family proteins and aggrephagy. A mutation in CCT2 (T400P) has been found in Leber congenital amaurosis/LCA. This mutation impairs Atg8-family protein association and aggrephagy via reducing CCT2 monomer production resulting in aggregation-prone protein buildup. Thus, the transition of CCT2 function in the two lines of cellular defense for proteostasis is determined by both complex and monomer formation (Ma et al., 2022b).

**Figure 4:**
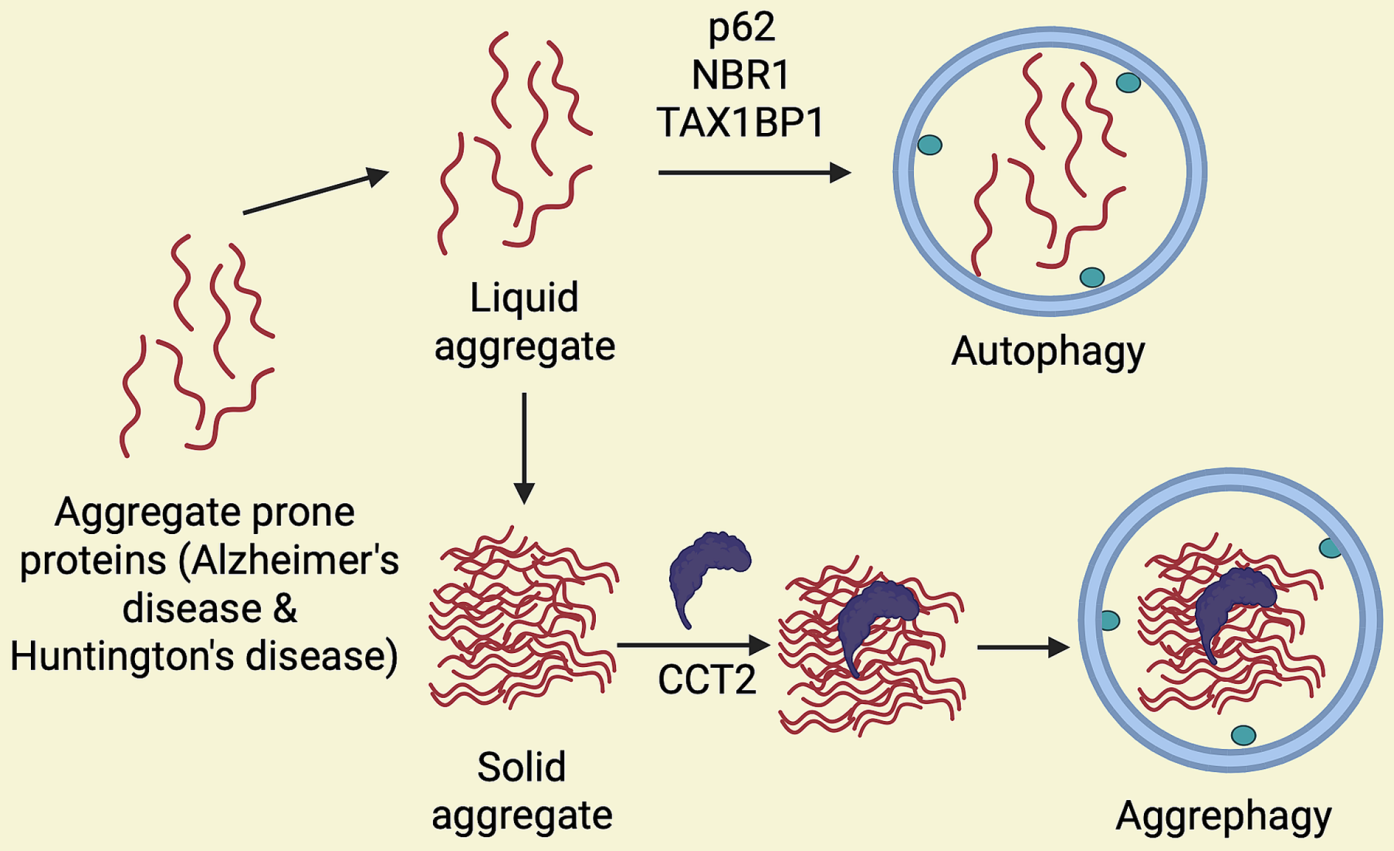
Role of CCT2 in aggrephagy. Aggregate-prone proteins undergo liquid aggregation that is degraded by autophagy in the presence of ubiquitin-binding receptors (p62, NBR1, and TAX1BP1). Liquid proteins transition into solid aggregate and CCT2 binds them to facilitate aggrephagy and license from BioRender.com.

A critical liquid–liquid phase separation stage occurs when misfolded proteins transit into solid protein aggregates (Babinchak and Surewicz, 2020). Liquid protein aggregates are specifically broken down by autophagy. In contrast, solid protein clumps were thought to be less receptive to autophagic clearance, and they were eventually stored within cells as inclusions to reduce cellular injury (Zhang et al., 2018). Using genetic engineering and photobleaching methods, Luo et al. found that ubiquitin-binding receptors (p62, NBR1, TAX1BP1) and CCT2 differentially target aggregates with varying fluidities for degradation (Luo et al., 2024). CCT2 is more likely to interact with less mobile, solid aggregates, while the autophagic clearance of ubiquitin-binding receptors is mediated by their preference for fluid protein aggregates or liquid-like assemblies of proteins (Luo et al., 2024) (Figure 4). Given its capacity to selectively target solid aggregates, CCT2 may play an important role in neurodegenerative diseases, where insoluble protein aggregates build up.

In closing, as a molecular chaperone, CCT2 works with other CCT subunits to promote proper protein folding and protect cellular protein stability in the early phases of protein homeostasis imbalance. As the imbalance worsens, CCT2 separates from the CCT complex and takes on a unique function as an autophagy receptor, causing protein aggregates, especially solid aggregates, to accumulate inside cells. Cellular proteostasis is further reinforced when CCT2 binds to these solid aggregates and guides them toward autophagosomes, which coordinates their degradation through the autophagic route (Luo et al., 2024) (Figure 4).

### TRiC/CCT as therapeutic target in proteinopathies

The TRiC/CCT chaperonin complex is emerging as a promising therapeutic target across multiple neurodegenerative diseases characterized by protein misfolding and aggregation (Ghozlan et al., 2022). In Huntington’s disease, TRiC/CCT plays critical roles in buffering the toxicity of mutant huntingtin (mHTT), with evidence showing that TRiC can bind and stabilize mHTT to prevent its conversion into toxic oligomers and aggregates (Tam et al., 2006). Subunit-specific studies have highlighted the TRiC’s modularity in folding activity (Kitamura et al., 2006; Spiess et al., 2004), making the complex a promising therapeutic target. Indirect enhancement of TRiC/CCT function could be achieved through pharmacological agents like Arimoclomol, a co-inducer of heat shock proteins, which may synergize with TRiC to suppress or alleviate mHTT toxicity (Kieran et al., 2004). Likewise, compounds that activate the heat shock response (HSR) or unfolded protein response (UPR), including geldanamycin and celastrol, can increase cell folding capacity (Sittler et al., 2001; Westerheide et al., 2004) and could support the activity of TRiC in suppressing mHTT aggregation. While no direct activators of TRiC/CCT have yet entered clinical development, high-throughput screens are underway to identify allosteric activators of TRiC that enhance its ATPase-driven substrate cycling (Joachimiak et al., 2014).

TRiC also plays a protective role against Aβ toxicity. Overexpression of specific CCT subunits has been shown to suppress Aβ-induced paralysis in animal models, highlighting the ability of TRiC to mitigate Aβ insults (Khabirova et al., 2014). Although no direct activators of TRiC are currently available in the clinic, compounds such as 4-phenylbutyrate (4-PBA) and trehalose function as chemical chaperones that stabilize protein conformation and indirectly enhance chaperonin activity (Cortez and Sim, 2014). These compounds have shown efficacy in models of Aβ toxicity and may act synergistically with endogenous CCT subunits to alleviate Aβ aggregation (Ricobaraza et al., 2011; Wiley et al., 2011). These strategies might be particularly effective in preventing the intracellular misfolding and oligomerization of Aβ before it is secreted. However, the case of extracellular Aβ requires special consideration, as its regulation by cytosolic chaperones may involve indirect or poorly understood mechanisms. Interestingly, evidence suggests that TRiC subunits, particularly CCT2, may also function outside the cell. According to Gene Cards *COMPARTMENTS* data, CCT2 is found in extracellular locations and is among the top 50 most abundant proteins in exosome preparations (Basso and Bonetto, 2016), this raises the possibility that CCT2 may influence extracellular Aβ dynamics via exosome-mediated secretion. Thus, therapeutic strategies aimed at enhancing the extracellular release of TRiC components, particularly CCT2, could represent a novel approach to target extracellular Aβ pathology. Given that CCT2 levels are significantly reduced in AD brains (Minjarez et al., 2016), restoring or maintaining its expression in early disease stages may have meaningful therapeutic impact. Importantly, viral vectors such as AAV9 could deliver CCT subunit genes selectively to affected brain regions. In addition, emerging tools such as CRISPRa (CRISPR activation) systems can be used to transcriptionally activate endogenous CCT subunit genes, avoiding the risks of gene insertion (Savell et al., 2019). These sophisticated approaches offer a next-generation solution to potentially restore dysregulation of TRiC subunits in Aβ-affected neurons.

In tauopathies, TRiC subunits such as CCT2, CCT3, CCT5, and CCT7 have been shown to inhibit tau aggregation (Ben-Maimon et al., 2025; Chen et al., 2018; Ma et al., 2022b) and we show here the dramatic CCT2-mediated protection against tau toxicity in transgenic flies (Figure 3). However, since multiple CCT subunits have been found associated with phosphorylated tau in proximity proteomics (Morderer et al., 2025), it is unclear if the entire holo-complex is involved or if pathological tau interacts specifically with certain subunits. Regardless of this, pharmacologic enhancers like Arimoclomol could potentiate the activity of endogenous CCT subunits, mitigating tau misfolding (Kampinga and Bergink, 2016). Also, as discussed above for Aβ toxicity, the delivery of TRiC components to tau-vulnerable regions of the brain via viral vectors or CRISPRa strategies holds potential to halt or reverse tau pathology in preclinical models.

Collectively, all these strategies highlight the growing relevance of the TRiC/CCT complex as a disease-modifying platform for neurodegenerative disorders driven by protein aggregation. However, given that aberrant regulation of CCT subunits has also been implicated in cancer cell proliferation (Ghozlan et al., 2022), future research must prioritize understanding the context-dependent control of TRiC/CCT activity to ensure both efficacy and safety in clinical applications.

### Open questions on the role of TRiC/CCT complex in protein misfolding and neurodegeneration

1. Mechanistic Insights:
  - What are the specific mechanisms by which TRiC/CCT complex subunits, particularly CCT2, modulate the folding and aggregation of misfolded proteins associated with neurodegenerative diseases?
  - What are the molecular triggers mediating the transition of CCT2 from chaperone subunit to autophagy receptor?
  - What mechanisms enable CCT2 to selectively recognize and target solid versus liquid protein aggregates for degradation?
  - How do post-translational modifications of TRiC/CCT subunits influence their interactions with misfolded proteins and their ability to prevent or promote aggregation?
2. Subunit Specificity:
  - What is the extent of subunit specificity within the TRiC/CCT complex regarding its interactions with different misfolded proteins implicated in neurodegenerative diseases?
  - Do individual subunits of the TRiC/CCT complex exhibit differential effects on protein folding and aggregation kinetics, and if so, what are the underlying molecular determinants?
3. Cellular Context:
  - How does the cellular environment, including factors such as pH and cellular stress, influence the function of TRiC/CCT subunits in protein folding and aggregation?
  - Are there cell type-specific differences in the expression or activity of TRiC/CCT subunits that contribute to the tissue-specific patterns of protein aggregation observed in neurodegenerative diseases?
4. Disease Progression:
  - What are the temporal dynamics of TRiC/CCT complex dysfunction during the progression of neurodegenerative diseases, and how do these dynamics correlate with changes in protein misfolding and aggregation?
  - Do alterations in TRiC/CCT complex function precede or coincide with the onset of protein aggregation and neurodegenerative pathology, and can they serve as early biomarkers or therapeutic targets?
5. Therapeutic Interventions:
  - Can targeted modulation of TRiC/CCT complex activity, either through small molecule potentiators or genetic manipulation mitigate protein aggregation in neurodegenerative diseases?
  - What are the potential off-target effects of pharmacological manipulation of TRiC/CCT complex function, and how can these be minimized to ensure therapeutic efficacy and safety?

Addressing these unknown or open questions will be crucial to advance our understanding on the role of the TRiC/CCT complex subunits in protein misfolding and neurodegenerative diseases, ultimately facilitating the development of novel therapeutic strategies for these devastating disorders.

## Concluding remarks

The chaperonin TRiC/CCT complex plays a dual role in protein folding and aggregation, with implications for the pathogenesis of neurodegenerative diseases. Animal and cellular models of neurodegenerative conditions have shown that impairments in this complex, along with the increased accumulation of misfolded proteins, lead to more severe phenotypes, reflecting the sophisticated interplay between the protein folding machinery and neurodegeneration. Among all TRiC subunits, CCT2 has been implicated in modulating the aggregation of various proteins associated with Alzheimer’s disease, tauopathies, and polyglutamine diseases. Its non-canonical role as an autophagy receptor responsible for clearing solid protein aggregates has significantly advanced our understanding of aggrephagy. It is unclear at present if other subunits or accessory factors are also involved in this process. Therefore, further research into the molecular mechanisms underlying TRiC function and its complex role in protein aggregation may pave the way for the development of novel therapeutic interventions targeting neurodegenerative diseases. In this regard, we strongly believe that studies focusing on restoring or enhancing TRiC’s folding capabilities should be an area of priority to alleviate disease symptoms and slow down neurodegeneration.
